# The causal association between Celiac disease and Ankylosing spondylitis: a two-sample Mendelian randomization analysis

**DOI:** 10.1186/s41927-026-00679-z

**Published:** 2026-07-31

**Authors:** Meiqi Chen, Xiang Gao

**Affiliations:** Department of Basic Medical Sciences, Qinghai University Medical College, Xining, Qinghai 810016 China

**Keywords:** Mendelian randomization, Celiac disease, Ankylosing spondylitis, gut-joint axis, seronegative arthritis, autoimmune diseases

## Abstract

**Objective:**

The concept of the gut-joint axis is often mentioned when exploring the pathogenesis of Ankylosing spondylitis (AS). This study aims to explore whether there is a causal association between Celiac disease (CeD) and AS.

**Method:**

Two-sample Mendelian randomization (MR) analysis was conducted using the CeD dataset (the largest pure CeD phenotype dataset in the IEU OpenGWAS database at the time of data retrieval, *n* = 24,269) and the AS dataset (strictly defined, from the IEU OpenGWAS database, *n* = 218,030). Inverse Variance Weighting (IVW) was adopted as the primary MR analysis method, supplemented by Weighted Median Estimator, MR-Egger regression, Simple Mode, and Weighted Mode to comprehensively evaluate the causal association between CeD and AS. Additionally, horizontal pleiotropy, heterogeneity, and leave-one-out analysis were performed to further evaluate the robustness and reliability of the results.

**Results:**

IVW analysis showed a positive causal effect of genetically predicted CeD on AS (OR: 1.435, 95% CI: 1.210–1.702, *p* = 3.34E-05), and the other four analytical methods also yielded consistent causal effect evaluation results. No significant horizontal pleiotropy or heterogeneity was observed in this study.

**Conclusion:**

MR analysis indicates that CeD may be a potential risk factor for AS, providing genetic evidence for the increased risk of AS in individuals with a genetic predisposition to CeD.

## Introduction

Ankylosing Spondylitis (AS) is a chronic, slowly progressive inflammatory autoimmune disease that primarily affects the axial skeleton, sacroiliac joints, and adjacent supporting soft tissues [1, 2]. Clinical features include pain and stiffness in the sacroiliac joints and/or lumbodorsal region, vertebral fusion, and decreased spinal mobility; the “Bamboo Spine” formed in the advanced stage may lead to disability [3]. Due to differences in geographic regions, study populations, and other factors, the prevalence of AS varies among different studies, which ranges approximately from 1‰ to 3‰ in the general population [4]. The etiology of AS is highly complex, potentially involving genetic, infectious, immunological, mechanical stress, endocrine, and other factors, among which genetic factors are considered primary. Although the positive rate of the HLA-B27 marker in patients with AS is as high as 80–96% [5, 6], it is estimated that only about 1–8% of the HLA-B27-positive population will develop AS [1, 6, 7]. HLA-B27 only increases the risk of developing AS but is not sufficient to cause the disease per se, which points to other non-HLA-B27 factors influencing the occurrence of AS. The early symptoms of AS have low specificity and can be easily confused with those of other diseases; therefore, it is often not diagnosed until 5 to 10 years after the onset. Considering the high risk of disability in advanced AS and the limited clinical efficacy of current drugs, it is crucial to investigate the risk factors of AS to promote early screening and intervention.

In studies on HLA-B27 transgenic rats, Taurog et al. reported that a germfree state can effectively prevent the development of inflammatory diseases in the gut and joint [8]. Furthermore, De Vos et al. reported that 56% of AS patients had inflammatory lesions during ileocolonoscopy [9]. The close anatomical proximity of the lumbar spine, sacrum, and sacroiliac joints to the gut enables the transmission of localized inflammatory signals from the gut more readily to these skeletal structures via the lymphatic and/or circulatory systems. Continuous exposure of the gut to exogenous antigens drives sustained activation of local immune cells; these gut-derived activated immune cells likely provide the effector cytokines required for AS initiation [10]. A compelling hypothesis posits that localized gut inflammation increases gut permeability and impairs oral tolerance, thereby allowing exogenous antigens to enter the systemic circulation and potentially trigger joint inflammation in genetically predisposed patients [9].

Celiac disease (CeD) is an autoimmune disorder triggered by gluten intolerance, with a global prevalence of approximately 1% [11, 12]. Once gluten enters the intestinal lumen, its digestive products permeate into the lamina propria and trigger an adaptive immune reaction, an innate immune response, as well as an autoimmune disorder in individuals with gluten intolerance [11]. Currently, several Mendelian randomization (MR) studies have reported potential causal associations between CeD and a number of autoimmune diseases, including but not limited to systemic lupus erythematosus (SLE) [13], psoriasis [14], and inflammatory bowel disease (IBD) [15]. However, there are few reports on the association between CeD and AS, and existing observational studies suggest that there is no significant association between the two [16]. Given that traditional observational studies are often subject to small sample sizes, confounding factors, and reverse causation, the robustness of their causal inference results is difficult to guarantee. This study will employ the MR method to investigate whether there is a potential causal association between CeD and AS.

MR analysis is a method for causal inference that utilizes single nucleotide polymorphisms (SNPs) highly correlated with the exposure as instrumental variables (IVs) to substitute for the exposure and infer the causal relationship between the exposure and the outcome. According to Mendel’s law of independent assortment (the second law), alleles biologically associated with the exposure are randomly allocated during gametogenesis and the genotype remains unchanged after conception, which is typically independent of external environmental risk factors and temporally precedes both the risk factors and the occurrence and progression of the disease [17]. Based on the above theory, MR analysis avoids interference from confounding factors such as environment and behavior, and eliminates the issue of reverse causation. By utilizing genetic predictors to conduct causal inference, MR analysis overcomes the inherent limitations of traditional observational studies and randomized controlled trials (RCTs) [18–20]. Therefore, we conducted a two-sample MR analysis on data from large-scale genome-wide association studies (GWAS) to explore the potential causal association between CeD and AS. This study aims to provide important evidence that CeD may be a risk factor for AS.

## Methods

### Data source

The data used in this study were obtained from the IEU OpenGWAS (Integrative Epidemiology Unit Open Genome-Wide Association Study Project; official website: https://gwas.mrcieu.ac.uk/). The data in this database were collected after obtaining ethical approval from relevant responsible authorities and informed consent from participants. Therefore, this study is considered exempt from additional ethical approval, as it is entirely based on data from a public database. This study obtained two datasets from the IEU OpenGWAS database: the largest publicly available pure CeD phenotype dataset at the time of data retrieval (ieu-a-1058; *N* = 24,269, 12,041 cases / 12,228 controls) [21] and the strictly defined AS dataset (finn-b-M13_ANKYLOSPON_STRICT; *N* = 218,030, 599 cases / 217,431 controls). To minimize the impact of population stratification, the study subjects were predominantly of European ancestry, regardless of sex. Additionally, the exposure and outcome samples were derived from independent study cohorts and had completely non-overlapping recruitment regions; accordingly, significant sample overlap sufficient to bias the causal effect estimates is unlikely.

### Selection of instrumental variables

The IVs must satisfy the following three key assumptions: (1) SNPs are strongly associated with the exposure (CeD); (2) SNPs are not associated with the outcome (AS); and (3) SNPs are not associated with any confounding factors. In this study, exposure-associated SNPs were extracted from the IEU OpenGWAS database using R Studio. To identify SNPs strongly associated with the exposure, a significance threshold of *p* < 5 × 10⁻⁸ was applied. Linkage disequilibrium (LD) was then pruned by setting r² < 0.001 and a genetic distance threshold of 10,000 kb. After selecting SNPs strongly associated with CeD, we first took their intersection with SNPs from the AS dataset, then aligned the effect alleles, and removed palindromic SNPs with ambiguous strand orientation as well as incompatible SNPs. Furthermore, via the LDtrait web tool (https://ldlink.nci.nih.gov/?tab=ldtrait), we manually screened and excluded SNPs associated with chronic inflammatory diseases (including AS), autoimmune traits (pleiotropy), or multiple osteoarthritic symptoms to control for horizontal pleiotropy bias. Finally, we calculated the F-statistic to eliminate weak IVs, that is, only retaining SNPs with an F-statistic > 10 [22, 23].

### Mendelian randomization analysis

This study employed five methods—Inverse Variance Weighting (IVW), Weighted Median Estimator (WME), MR-Egger regression, simple mode (SM), and weighted mode (WM)—to analyze the causal association between CeD and AS. When the core assumptions of MR are satisfied, IVW has been proven to be one of the most efficient and unbiased methods; therefore, it was selected as the primary analytical method in this study [23, 24]. IVW is based on the premise that all genetic variants used are valid IVs, meaning they satisfy the three key assumptions of MR (specifically, that all genetic variants exhibit no horizontal pleiotropy). In contrast, MR-Egger regression does not require IVs to strictly satisfy the three assumptions (i.e., it allows for the presence of horizontal pleiotropy across all genetic variants) but provides consistent estimates of the causal effect under weaker assumptions [25, 26]. WME offers a compromise approach to address the issue of having some invalid IVs in MR analysis. It can provide a consistent estimate of the causal effect even when up to 50% of the information comes from invalid IVs [27], thereby complementing other analytical methods.

### Sensitivity analysis

Heterogeneity was assessed using Cochran’s Q statistic, with *p* > 0.05 suggesting no substantial heterogeneity and *p* < 0.05 indicating its presence. If no heterogeneity was detected in the results, the IVW fixed-effects model was routinely applied; otherwise, the IVW random-effects model was employed. To ensure the stability and reliability of causal estimates, potential influential SNPs were identified and removed using either MR-Presso or leave-one-out analysis. For sensitivity analysis, the leave-one-out method was performed by systematically excluding each SNP one at a time, and calculating the combined effect of the remaining SNPs, thereby evaluating whether any individual SNP disproportionately influenced the exposure-outcome association. The MR-Egger intercept test was further conducted to examine potential horizontal pleiotropy among the IVs (SNPs), thereby assessing the overall robustness of the MR analysis.

### Statistical analysis

This two-sample MR analysis was performed using the TwoSampleMR package in R software (version 4.2.1).

## Results

### Selected instrumental variables

Based on the pre-specified criteria (*p* < 5 × 10⁻⁸, r² < 0.001, clumping distance = 10,000 kb), 15 SNPs strongly associated with CeD were selected as potential IVs from the exposure dataset. After harmonization, all 15 SNPs were successfully matched; no palindromic SNPs were identified, and no proxy SNPs were used. We then conducted manual screening using the LDtrait web tool, and 5 SNPs were removed based on the database annotations available at the time of retrieval: rs10752747 and rs13119723, which were associated with chronic inflammatory diseases (including AS); rs11979905 and rs10892258, which were linked to “autoimmune traits (pleiotropy)”; rs931, which was associated with multiple osteoarthritic symptoms. Leave-one-out analysis showed that two additional SNPs (rs130078, rs13198474) were outlier loci that drove significant heterogeneity and disproportionately influenced the overall robustness; their exclusion effectively controlled heterogeneity and yielded a final set of 8 SNPs as IVs for subsequent MR analysis (details in Table 1). The F statistics for all these IVs were well above 10 (see Table 1), confirming strong predictive power for the exposure and indicating no weak instrument bias, which enhances the reliability of the causal estimates.

**Table 1:** Detailed information of selected instrumental variables

SNP	Chr.	EA/OA	EAF	beta	se	*p*	*R* ^2^	F
rs12527282	6	T/C	0.280	-0.155	0.021	1.69E-13	0.010	235.932
rs12663317	6	C/A	0.085	-0.616	0.043	3.53E-47	0.059	1515.358
rs13030124	2	A/G	0.450	-0.105	0.019	2.40E-08	0.005	133.524
rs2441467	2	C/T	0.418	0.105	0.019	1.70E-08	0.005	131.558
rs6498114	16	T/G	0.756	-0.131	0.021	5.83E-10	0.006	154.684
rs6926336	6	C/T	0.022	-0.812	0.080	6.12E-24	0.028	705.994
rs7162232	15	A/G	0.681	-0.117	0.020	7.97E-09	0.006	144.856
rs9296009	6	T/A	0.234	-0.954	0.029	1.00E-200	0.326	11755.600

Abbreviation: SNP, single nucleotide polymorphism; Chr., chromosome; EA/OA, Effect Allele/Other Allele, EAF, Effect Allele Frequency

### Mendelian randomization analysis

**Fig. 1:**
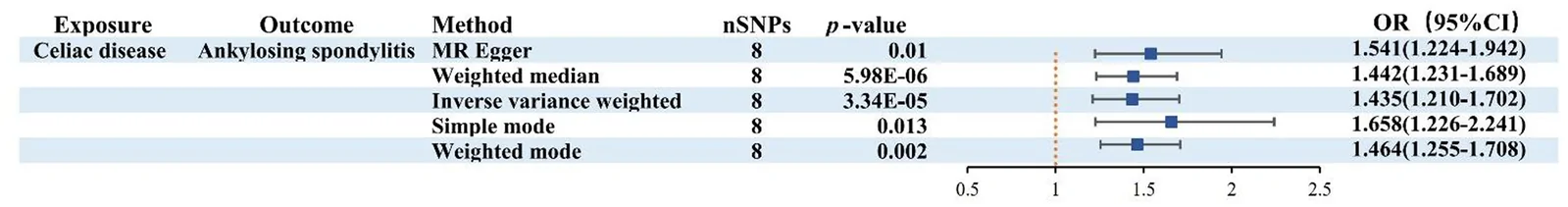
The result plot of the MR causal association analysis between CeD and AS

**Fig. 2:**
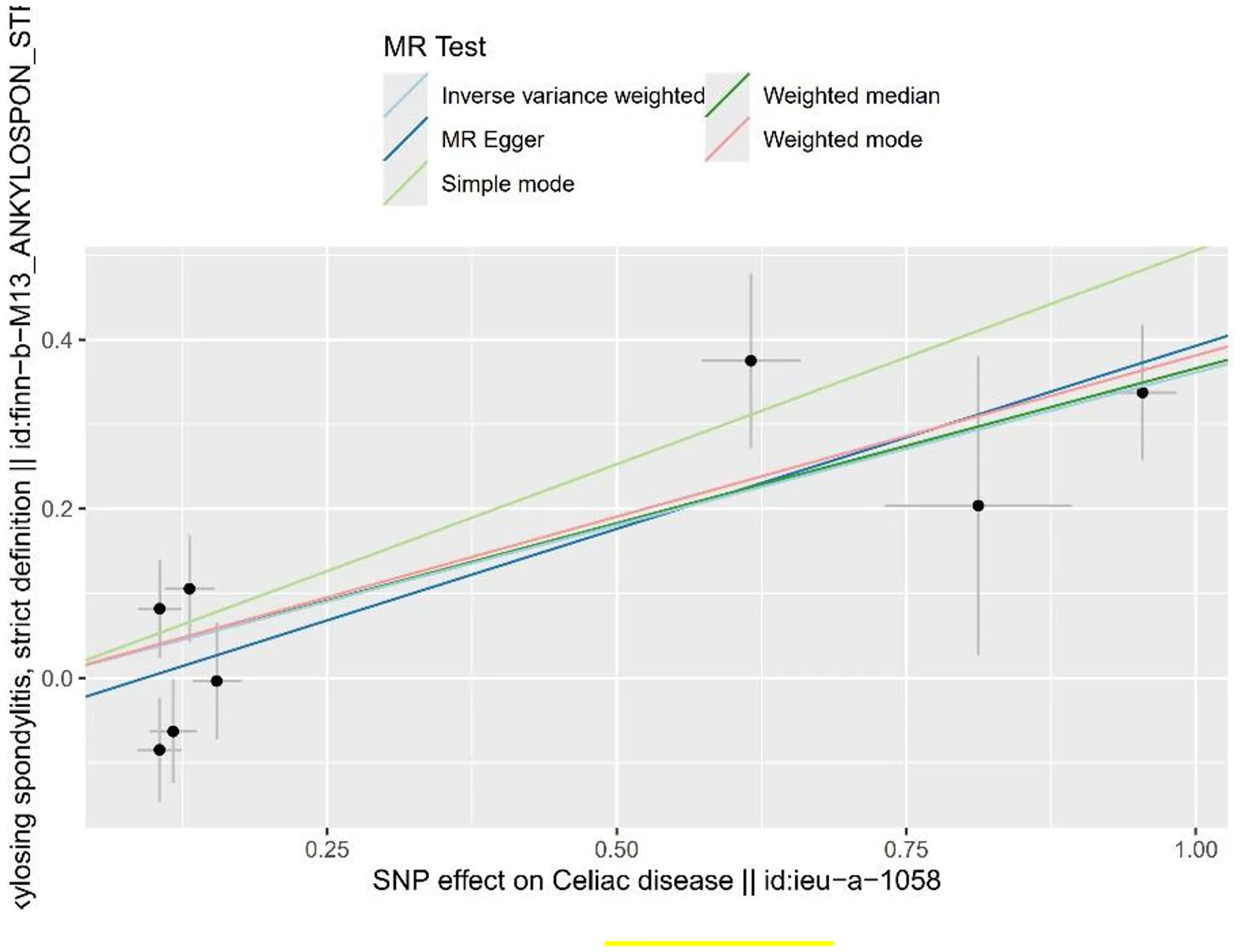
The scatter plot for the causal effect of genetically predicted CeD on AS

The initial MR analysis showed a significant causal effect of genetically predicted CeD on AS (IVW OR: 1.435, 95%CI: 1.210–1.702, *p* = 3.34E-05; WME OR: 1.442, 95%CI: 1.231–1.689, *p* = 5.98E-06; MR-Egger OR: 1.541, 95%CI: 1.224–1.942, *p* = 0.010). This causal effect was consistently supported by estimates from both the WM and the SM. Overall, results from all five analytical methods indicated a positive causal effect of CeD on AS. Detailed MR estimates are shown in Fig. 1, and the scatter plot of MR analysis is presented in Fig. 2.

### Sensitivity analysis

**Fig. 3:**
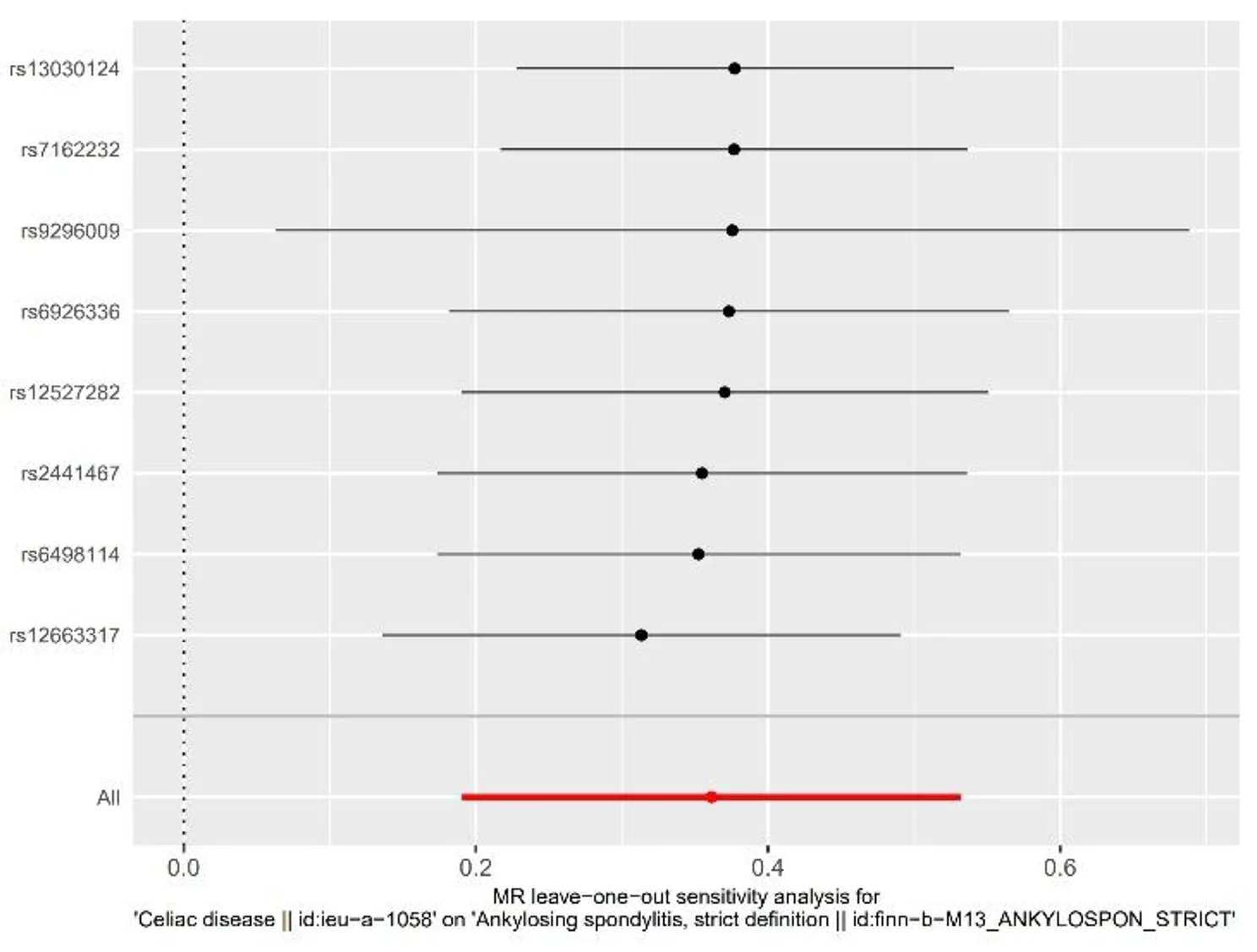
The leave-one-out plot for the causal effect of genetically predicted CeD on AS

**Fig. 4:**
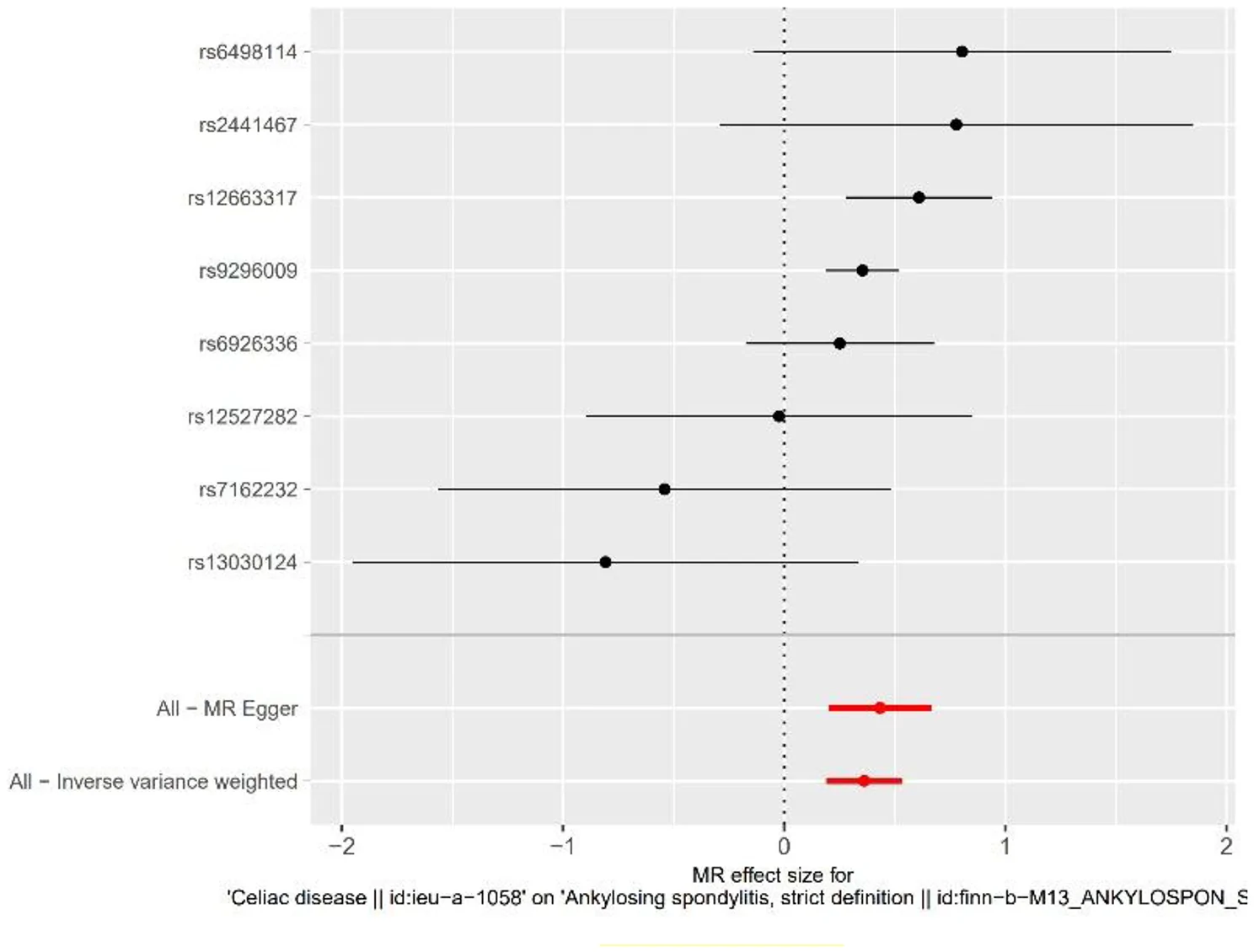
The forest plot for the causal effect of genetically predicted CeD on AS

Cochran’s Q test showed *p* > 0.05, indicating that no significant heterogeneity was detected among the IVs included in this analysis (see Table 2 for details). The intercept of MR-Egger regression was extremely close to zero with *p* > 0.05, suggesting no evidence of significant horizontal pleiotropy—i.e., the exclusion restriction assumption was not violated (see Table 2 for details). Leave-one-out sensitivity analysis showed that the genetically predicted causal association exhibited good robustness (Fig. 3). The forest plot is presented in Fig. 4.

**Table 2:** Test results of heterogeneity and pleiotropy for instrumental variables

Exposure	Outcome	No. IVs	IVW			MR-Egger			
			Q statistic	*p*-value		Q statistic	*p*-value	Intercept	*p*-value
CeD	AS	8	11.650	0.113		10.227	0.115	-0.040	0.396

Abbreviation: CeD, Celiac disease; AS, Ankylosing Spondylitis; No. IVs, number of instrumental variables; IVW, Inverse Variance Weighting

## Discussion

This study employed MR to investigate the potential causal association between CeD and AS. All five MR methods (IVW, MR-Egger, WME, SM, and WM) consistently indicated a significant positive causal association between genetically predicted CeD and AS. Sensitivity analysis supported the robustness of the study findings. These findings were not significantly influenced by any single SNP, thus enhancing the reliability of the conclusions. The results of this two-sample MR study provide genetic evidence that CeD may be a potential risk factor for AS.

Some existing studies indicate a higher prevalence of other autoimmune diseases among CeD patients compared to the g eneral population [28–34]. In 1995, Usai et al. performed bone scintigraphy on 22 adult CeD patients and detected sacroiliitis in approximately 63.6% of them [35]. Notably, sacroiliitis is considered the initial pathological manifestation of AS [3, 36]. In fact, the joints involved in CeD and AS almost completely overlap. The most frequent sites of skeletal involvement in CeD are the spine (particularly the lumbar spine), the sacroiliac joints, the hip joints, and the knee joints, among others [16, 37]. Furthermore, the arthritis of CeD closely resembles other enteropathic arthritides, which are classified as seronegative spondyloarthropathies [37]—the same disease family to which AS belongs. Two other studies have detected CeD‑related autoantibodies in AS patients: anti‑gliadin antibodies (AGA) were positive in about 36.7% of cases [38], and human anti‑tissue transglutaminase immunoglobulin A (htTG‑IgA) in 18.4% [39]. The intestinal histopathological features in patients with AS are also remarkably similar to those in CeD, including crypt hyperplasia, villous atrophy, and increased infiltration of inflammatory cells such as lymphocytes and plasma cells in the lamina propria [40]. Moreover, like patients with CeD, those with AS also exhibit increased gut permeability [41, 42]. However, observational studies have not detected cases of AS that are attributable to CeD [16]. Similarly, Iqbal et al. reported that the prevalence of spondyloarthritis in CeD is very low, approximating the prevalence of AS in the general population [32]. This may be attributed to the fact that traditional observational studies are often limited by insufficient sample size, coupled with the interference of confounding factors and reverse causation, leading to the conclusion of a negative causal association.

This study utilized a two-sample MR approach to uncover the potential causal association between CeD and AS. By strictly controlling the representativeness of IVs and relying on their innate random distribution across “each observation group”, MR analysis is the method closest to RCTs and is capable of providing a valid assessment of causal associations. Compared to RCTs, MR analysis is more straightforward to implement, has a shorter duration, avoids ethical issues, and is less costly. Relative to observational studies, it leverages larger sample sizes and more effectively minimizes confounding and reverse causation, and is thus more likely to yield unbiased estimates. The drafting of this manuscript spanned an extended period, and prior to its finalization, a similarly focused study appeared [43]. By comparison, our study employed the largest publicly available pure CeD phenotype dataset at the time of data retrieval and the strictly defined AS dataset, yielding higher statistical power for causal association analysis. This is one of the key strengths of our study. Furthermore, rigorous control of heterogeneity and horizontal pleiotropy, together with consistent causal estimates across five complementary analytical methods, provides robust support for genetically predicted CeD as a potential risk factor for AS, representing a further key strength of our study. Of note, after careful consideration of multiple factors, the present study elected not to conduct reverse MR analysis, for two primary reasons. First, in terms of clinical onset temporality, the clinical onset of CeD generally precedes that of AS. Using a later-onset disease as the exposure to infer its causal effect on an earlier-onset outcome violates the genuine temporal logic that cause must precede effect, rendering the analytical results not amenable to a genuine causal interpretation. Second, from the perspective of clinical translational value, given its later onset, insidious presentation, and delayed diagnosis, AS cannot provide valuable clinical guidance for the early screening and complication prevention and management of CeD.

This study has several limitations. First, almost all data were derived from European populations. While this reduces bias from population stratification, it may limit the generalizability of the findings to other ethnic groups. Second, given the complexity and unknown factors in clinical contexts and in the genetic instruments (SNPs), it is unlikely that all confounding factors could be excluded. Finally, the MR results may be influenced by dataset updates, R software iterations, and methodological improvements in MR analysis, which could affect the robustness of the causal estimates. Given these limitations, the findings of this study should be interpreted cautiously.

As is well known, RCTs represent the “gold standard” for causal inference, but their major limitation may lie in ethical constraints (as when the exposure is a harmful factor) that prevent such trials from being conducted. Observational studies, being susceptible to confounding factors and reverse causation, seldom establish direct causal relationships. In this study, we employed two‑sample MR analysis to emulate an RCT for assessing the causal link between exposure and outcome. This approach circumvents ethical issues and reverse causality while minimizing confounding. Our findings support genetically predicted CeD as a potential risk factor for AS, a conclusion bolstered by the consistency across five MR methods, pleiotropy tests, and sensitivity analyses.

The marked delay in diagnosing AS and the substantial long-term disability risk associated with the disease have long been a major dilemma for both clinicians and patients. Based on our findings, incorporating AS-related assessments into the clinical management approach for CeD patients with spinal or osteoarticular symptoms holds certain clinical guiding value for the early diagnosis and intervention of AS. In the future, prospective cohort studies may be needed to validate the present findings. Further research should explore the biological mechanisms linking CeD and AS, which will not only advance the understanding of the gut–joint axis in AS but also provide a theoretical basis for identifying clinical intervention targets.

## Data Availability

Publicly available datasets were obtained from the IEU Open GWAS, available at https://gwas.mrcieu.ac.uk.
